# {1,5,9-Tris[(2*S*)-2-hydroxy­prop­yl]-1,5,9-triaza­cyclo­dodeca­ne}zinc(II) dinitrate monohydrate

**DOI:** 10.1107/S1600536810006410

**Published:** 2010-02-24

**Authors:** Christoph E. Strasser, Jimmy E. Y. Sumani, Helgard G. Raubenheimer, Robert C. Luckay

**Affiliations:** a Department of Chemistry and Polymer Science, University of Stellenbosch, Private Bag X1, Matieland, 7602, South Africa

## Abstract

In the title compound, [Zn(C_18_H_39_N_3_O_3_)](NO_3_)_2_·H_2_O, the coordination geometry around the central Zn^II^ atom is distorted octa­hedral. The hydroxyl groups in the macrocyclic ligand and water mol­ecules are engaged in O—H⋯O hydrogen bonding, which forms two-dimensional corrugated sheets comprising 34-membered rings. Neighbouring sheets are connected by C—H⋯O inter­actions.

## Related literature

For the synthesis, see: Richman & Atkins (1974 ▶); Sabatini & Fabrizzi (1979 ▶). For background to aza­macrocycles, see: Skerlj *et al.* (2002 ▶). For the use of functionalised macrocycles in the synthesis of metal-chelating agents for medical applications, see: Sheng *et al.* (2007 ▶).
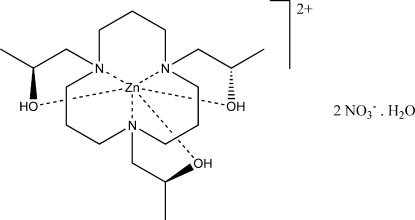


## Experimental

### 

#### Crystal data

[Zn(C_18_H_39_N_3_O_3_)](NO_3_)_2_·H_2_O
*M* *_r_* = 552.93
Orthorhombic, 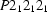

*a* = 10.1558 (10) Å
*b* = 15.4883 (15) Å
*c* = 15.7498 (15) Å
*V* = 2477.4 (4) Å^3^
*Z* = 4
Mo *K*α radiation
μ = 1.05 mm^−1^
*T* = 100 K
0.16 × 0.15 × 0.11 mm

#### Data collection

Bruker APEX CCD area-detector diffractometer
Absorption correction: multi-scan (*SADABS*; Bruker, 2002 ▶) *T*_min_ = 0.497, *T*_max_ = 0.890
14715 measured reflections
5102 independent reflections
4378 reflections with *I* > 2σ(*I*)
*R*_int_ = 0.042

#### Refinement

*R*[*F* ^2^ > 2σ(*F* ^2^)] = 0.048
*wR*(*F* ^2^) = 0.113
*S* = 1.05
5102 reflections
325 parameters
6 restraints
H atoms treated by a mixture of independent and constrained refinement
Δρ_max_ = 0.83 e Å^−3^
Δρ_min_ = −0.47 e Å^−3^
Absolute structure: Flack (1983 ▶), 2219 Friedel pairs
Flack parameter: −0.003 (15)

### 

Data collection: *SMART* (Bruker, 2002 ▶); cell refinement: *SAINT* (Bruker, 2003 ▶); data reduction: *SAINT*; program(s) used to solve structure: *SHELXS97* (Sheldrick, 2008 ▶); program(s) used to refine structure: *SHELXL97* (Sheldrick, 2008 ▶); molecular graphics: *X-SEED* (Atwood & Barbour, 2003 ▶; Barbour, 2001 ▶); software used to prepare material for publication: *SHELXL97*.

## Supplementary Material

Crystal structure: contains datablocks I, Global. DOI: 10.1107/S1600536810006410/ci5025sup1.cif

Structure factors: contains datablocks I. DOI: 10.1107/S1600536810006410/ci5025Isup2.hkl

Additional supplementary materials:  crystallographic information; 3D view; checkCIF report

## Figures and Tables

**Table 1:** Hydrogen-bond geometry (Å, °)

*D*—H⋯*A*	*D*—H	H⋯*A*	*D*⋯*A*	*D*—H⋯*A*
O1—H1⋯O10	0.86 (1)	1.80 (1)	2.652 (5)	171 (5)
O2—H2⋯O4	0.84 (1)	1.80 (2)	2.624 (5)	166 (5)
O3—H3⋯O6	0.85 (1)	2.06 (2)	2.853 (4)	156 (5)
O3—H3⋯O7	0.85 (1)	2.54 (3)	3.199 (4)	135 (4)
O4—H4*C*⋯O8^i^	0.85 (1)	1.86 (4)	2.641 (7)	153 (8)
O4—H4*D*⋯O5^ii^	0.84 (1)	1.86 (2)	2.689 (5)	170 (8)
C9—H9*B*⋯O10^iii^	0.99	2.39	3.184 (6)	137

Symmetry codes: (i) 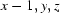; (ii) 
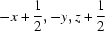
; (iii) 
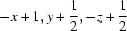
.

**Fig. 1.**
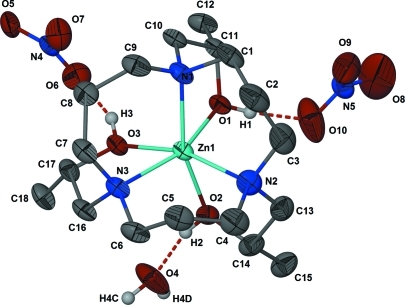
The asymmetric unit of the title compound. Displacement ellipsoids are drawn at the 50% probability level.

**Fig. 2.**
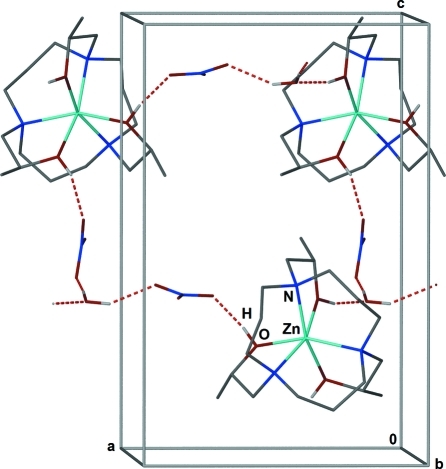
Part of the infinite sheet formed by hydrogen bonds (dashed lines).

## supplementary crystallographic information

### Comment

Azamacrocycles and some of their *N*-substituted derivatives are of synthetic interest due to their unique binding properties with metal ions. The addition of different pendant arms can enhance the selectivity of the azamacrocycle for a metal cation, depending on the cavity size and on the nature of the substitutents (Skerlj *et al.*, 2002). The design and synthesis of polyazamacrocycles bearing flexible pendant arms from the cyclic framework provides chemists with an opportunity to design macrocycles tailored for a specific function. This is especially the case because the pendant arms provide additional coordination sites for metal ions.

Functionalised macrocycles have been successfully employed in the synthesis of metal-chelating agents for medical applications owing to the kinetic inertness of the complexes which makes them resistant to decomplexation (Sheng *et al.*, 2007).

In the title compound (Fig. 1), the Zn^II^ atom is octahedrally coordinated. Small distortions cause the N—Zn—N angles to exceed 90° while the O—Zn—O angles measure less than 90°. The dicationic complex exhibits near *C*_3_-symmetry with exception of the propylene groups in which the central CH_2_ group adopts a different conformation in each sector.

The three hydroxyl groups are all engaged in hydrogen bonds. The O1—H1 and O3—H3 groups form hydrogen bonds (Table 1) with the two asymmetric nitrate counter anions and the O2—H2 group with the water molecule. This water molecule itself forms two hydrogen bonds with two other complexes. The hydrogen bonding results in the formation of two-dimensional corrugated sheets parallel to the *ac* plane. Three adjacent complex units are linked to form 34-membered rings which include ten hydrogen bonds (Fig. 2). Neighbouring sheets are loosely connected by C—H···O interactions involving one of the CH_2_ groups of the triazacyclododecane.

The configuration at C11, C14 and C17 is (*S*), resulting from utilisation of enantiopure (*S*)-methyloxirane in the synthetic process which is supported by anomalous scattering.

### Experimental

The chiral macrocyclic ligand 1,5,9-tris[(2*S*)-2-hydroxypropyl]-1,5,9-triazacyclododecane was prepared by treating three equivalents and a slight excess of (*S*)-methyloxirane with 1,5,9-triazacyclododecane in absolute ethanol. This solution was stirred at room temperature for 3 days. 1,5,9-Triazacyclododecane was prepared using a modified method of Richman & Atkins (1974) and Sabatini & Fabrizzi (1979).

One equivalent of Zn(NO_3_)_2_.6H_2_O was dissolved in ethanol at 333 K with stirring. The ligand was added to the solution and it was stirred overnight. The powder which formed was filtered off and dissolved in a small quantity of *N*,*N*-dimethylformamide whereupon diethyl ether vapour was slowly diffused into the solution in a sealed container. Single crystals were obtained the next day.

### Refinement

Alkyl H atoms were positioned geometrically (C–H = 1.00, 0.99 and 0.98 Å for CH, CH_2_ and CH_3_ groups, respectively) and allowed to ride on their parent atoms. Hydroxyl and water O–H distances were restrained to 0.85 (1) Å and additional restraint H4···H5 distance restraint of 1.37 (1) Å was applied to the water molecule. *U*_iso_(H) values were set at 1.2 times *U*_eq_(C,O) except for methyl groups where *U*_iso_(H) was set at 1.5 times *U*_eq_(C).

### Crystal data

**Table:**

[Zn(C_18_H_39_N_3_O_3_)](NO_3_)_2_·H_2_O	*F*(000) = 1176
*M**_r_* = 552.93	*D*_x_ = 1.482 Mg m^−^^3^
Orthorhombic, *P*2_1_2_1_2_1_	Mo *K*α radiation, λ = 0.71073 Å
Hall symbol: P 2ac 2ab	Cell parameters from 2897 reflections
*a* = 10.1558 (10) Å	θ = 2.4–21.4°
*b* = 15.4883 (15) Å	µ = 1.05 mm^−^^1^
*c* = 15.7498 (15) Å	*T* = 100 K
*V* = 2477.4 (4) Å^3^	Block, colourless
*Z* = 4	0.16 × 0.15 × 0.11 mm

### Data collection

**Table:**

Bruker APEX CCD area-detector diffractometer	5102 independent reflections
Radiation source: fine-focus sealed tube	4378 reflections with *I* > 2σ(*I*)
graphite	*R*_int_ = 0.042
ω–scans	θ_max_ = 26.4°, θ_min_ = 1.8°
Absorption correction: multi-scan (*SADABS*; Bruker, 2002)	*h* = −12→10
*T*_min_ = 0.497, *T*_max_ = 0.890	*k* = −19→12
14715 measured reflections	*l* = −18→19

### Refinement

**Table:**

Refinement on *F*^2^	Secondary atom site location: difference Fourier map
Least-squares matrix: full	Hydrogen site location: inferred from neighbouring sites
*R*[*F*^2^ > 2σ(*F*^2^)] = 0.048	H atoms treated by a mixture of independent and constrained refinement
*wR*(*F*^2^) = 0.113	*w* = 1/[σ^2^(*F*_o_^2^) + (0.0571*P*)^2^ + 0.2902*P*] where *P* = (*F*_o_^2^ + 2*F*_c_^2^)/3
*S* = 1.05	(Δ/σ)_max_ = 0.001
5102 reflections	Δρ_max_ = 0.83 e Å^−^^3^
325 parameters	Δρ_min_ = −0.47 e Å^−^^3^
6 restraints	Absolute structure: Flack (1983), 2219 Friedel pairs
Primary atom site location: structure-invariant direct methods	Flack parameter: −0.003 (15)

### Special details

**Table:**

Geometry. All esds (except the esd in the dihedral angle between two l.s. planes) are estimated using the full covariance matrix. The cell esds are taken into account individually in the estimation of esds in distances, angles and torsion angles; correlations between esds in cell parameters are only used when they are defined by crystal symmetry. An approximate (isotropic) treatment of cell esds is used for estimating esds involving l.s. planes.
Refinement. Refinement of *F*^2^ against ALL reflections. The weighted *R*-factor wR and goodness of fit *S* are based on *F*^2^, conventional *R*-factors *R* are based on *F*, with *F* set to zero for negative *F*^2^. The threshold expression of *F*^2^ > σ(*F*^2^) is used only for calculating *R*-factors(gt) etc. and is not relevant to the choice of reflections for refinement. *R*-factors based on *F*^2^ are statistically about twice as large as those based on *F*, and *R*- factors based on ALL data will be even larger.

### Fractional atomic coordinates and isotropic or equivalent isotropic displacement parameters (Å^2^)

**Table:**

	*x*	*y*	*z*	*U*_iso_*/*U*_eq_	
Zn1	0.36584 (4)	0.27731 (2)	0.26347 (3)	0.02956 (13)	
O1	0.5352 (2)	0.20359 (16)	0.24231 (17)	0.0361 (6)	
H1	0.579 (4)	0.180 (3)	0.282 (2)	0.054*	
O2	0.3145 (3)	0.17597 (18)	0.34919 (18)	0.0384 (7)	
H2	0.253 (4)	0.141 (3)	0.337 (3)	0.058*	
O3	0.2876 (3)	0.18319 (16)	0.16470 (18)	0.0335 (6)	
H3	0.333 (4)	0.163 (3)	0.124 (2)	0.050*	
N1	0.4853 (3)	0.3643 (2)	0.1868 (2)	0.0351 (8)	
N2	0.4049 (4)	0.3366 (2)	0.3861 (2)	0.0445 (9)	
N3	0.1732 (3)	0.32353 (18)	0.2361 (2)	0.0350 (7)	
C1	0.5873 (4)	0.4178 (3)	0.2327 (3)	0.0461 (11)	
H1A	0.6652	0.3811	0.2441	0.055*	
H1B	0.6158	0.4654	0.1949	0.055*	
C2	0.5404 (5)	0.4553 (3)	0.3145 (4)	0.0617 (15)	
H2A	0.6020	0.5013	0.3325	0.074*	
H2B	0.4530	0.4820	0.3055	0.074*	
C3	0.5301 (6)	0.3898 (3)	0.3834 (4)	0.0608 (14)	
H3A	0.5391	0.4200	0.4385	0.073*	
H3B	0.6056	0.3498	0.3781	0.073*	
C4	0.2927 (6)	0.3893 (3)	0.4213 (3)	0.0554 (13)	
H4A	0.3306	0.4377	0.4543	0.067*	
H4B	0.2431	0.3526	0.4616	0.067*	
C5	0.1959 (5)	0.4264 (3)	0.3581 (3)	0.0497 (12)	
H5A	0.1377	0.4675	0.3883	0.060*	
H5B	0.2457	0.4595	0.3150	0.060*	
C6	0.1110 (4)	0.3611 (3)	0.3131 (3)	0.0459 (11)	
H6A	0.0897	0.3138	0.3531	0.055*	
H6B	0.0272	0.3891	0.2965	0.055*	
C7	0.1678 (4)	0.3901 (3)	0.1664 (3)	0.0421 (11)	
H7A	0.0837	0.3826	0.1354	0.051*	
H7B	0.1656	0.4480	0.1931	0.051*	
C8	0.2779 (5)	0.3893 (3)	0.1023 (3)	0.0446 (11)	
H8A	0.2503	0.4233	0.0522	0.054*	
H8B	0.2923	0.3291	0.0834	0.054*	
C9	0.4068 (4)	0.4252 (3)	0.1350 (3)	0.0454 (12)	
H9A	0.4606	0.4437	0.0858	0.054*	
H9B	0.3880	0.4771	0.1696	0.054*	
C10	0.5550 (4)	0.3027 (2)	0.1298 (3)	0.0341 (9)	
H10A	0.6191	0.3348	0.0945	0.041*	
H10B	0.4903	0.2753	0.0913	0.041*	
C11	0.6259 (4)	0.2340 (2)	0.1788 (3)	0.0376 (9)	
H11	0.7049	0.2597	0.2072	0.045*	
C12	0.6688 (4)	0.1599 (3)	0.1224 (3)	0.0455 (11)	
H12A	0.7148	0.1165	0.1566	0.068*	
H12B	0.7281	0.1816	0.0781	0.068*	
H12C	0.5912	0.1336	0.0959	0.068*	
C13	0.4320 (5)	0.2628 (3)	0.4426 (3)	0.0487 (12)	
H13A	0.4376	0.2839	0.5018	0.058*	
H13B	0.5185	0.2377	0.4274	0.058*	
C14	0.3291 (5)	0.1933 (3)	0.4378 (3)	0.0445 (12)	
H14	0.2441	0.2158	0.4609	0.053*	
C15	0.3691 (6)	0.1138 (3)	0.4862 (3)	0.0534 (12)	
H15A	0.3016	0.0691	0.4794	0.080*	
H15B	0.3783	0.1280	0.5466	0.080*	
H15C	0.4533	0.0924	0.4643	0.080*	
C16	0.0934 (4)	0.2471 (3)	0.2103 (3)	0.0402 (11)	
H16A	0.0049	0.2669	0.1925	0.048*	
H16B	0.0821	0.2087	0.2600	0.048*	
C17	0.1541 (4)	0.1966 (2)	0.1393 (3)	0.0379 (10)	
H17	0.1519	0.2316	0.0860	0.046*	
C18	0.0816 (5)	0.1124 (3)	0.1254 (3)	0.0467 (11)	
H18A	0.1221	0.0810	0.0781	0.070*	
H18B	−0.0109	0.1245	0.1120	0.070*	
H18C	0.0865	0.0773	0.1771	0.070*	
O4	0.1292 (4)	0.0583 (3)	0.3391 (3)	0.0907 (15)	
H4C	0.048 (2)	0.071 (4)	0.335 (5)	0.136*	
H4D	0.136 (7)	0.009 (2)	0.360 (5)	0.136*	
N4	0.3691 (4)	0.1325 (2)	−0.0369 (2)	0.0342 (7)	
O5	0.3483 (3)	0.10720 (19)	−0.11152 (19)	0.0468 (7)	
O6	0.3717 (4)	0.08361 (19)	0.0223 (2)	0.0583 (9)	
O7	0.3868 (4)	0.21105 (19)	−0.0261 (2)	0.0575 (9)	
N5	0.7946 (4)	0.1494 (2)	0.3579 (2)	0.0411 (9)	
O8	0.8856 (6)	0.1023 (4)	0.3790 (4)	0.143 (3)	
O9	0.8147 (4)	0.2251 (3)	0.3530 (2)	0.0804 (13)	
O10	0.6844 (5)	0.1216 (3)	0.3546 (3)	0.1017 (19)	

### Atomic displacement parameters (Å^2^)

**Table:**

	*U*^11^	*U*^22^	*U*^33^	*U*^12^	*U*^13^	*U*^23^
Zn1	0.0250 (2)	0.0255 (2)	0.0382 (2)	−0.00091 (18)	0.00286 (18)	−0.00223 (18)
O1	0.0246 (13)	0.0392 (15)	0.0447 (16)	0.0011 (11)	0.0036 (12)	0.0023 (13)
O2	0.0355 (18)	0.0408 (17)	0.0388 (16)	−0.0051 (13)	0.0048 (13)	0.0042 (13)
O3	0.0261 (15)	0.0302 (15)	0.0442 (17)	−0.0022 (11)	0.0004 (12)	−0.0033 (12)
N1	0.0268 (18)	0.0218 (15)	0.057 (2)	−0.0059 (13)	0.0024 (16)	−0.0041 (15)
N2	0.049 (2)	0.0370 (19)	0.048 (2)	0.0009 (16)	0.0025 (17)	−0.0158 (17)
N3	0.0273 (17)	0.0231 (15)	0.055 (2)	0.0008 (12)	0.0035 (15)	0.0006 (15)
C1	0.033 (2)	0.031 (2)	0.075 (3)	−0.0117 (16)	0.005 (2)	−0.015 (2)
C2	0.048 (3)	0.053 (3)	0.084 (4)	−0.015 (2)	0.005 (3)	−0.029 (3)
C3	0.064 (4)	0.051 (3)	0.068 (3)	−0.014 (3)	−0.010 (3)	−0.021 (3)
C4	0.057 (3)	0.046 (3)	0.062 (3)	0.006 (2)	0.009 (3)	−0.019 (2)
C5	0.051 (3)	0.035 (2)	0.063 (3)	0.010 (2)	0.011 (2)	−0.011 (2)
C6	0.033 (3)	0.037 (2)	0.068 (3)	0.0029 (19)	0.015 (2)	−0.001 (2)
C7	0.032 (3)	0.028 (2)	0.067 (3)	0.0011 (17)	−0.001 (2)	0.004 (2)
C8	0.045 (3)	0.032 (2)	0.057 (3)	0.003 (2)	−0.001 (2)	0.013 (2)
C9	0.039 (3)	0.028 (2)	0.069 (3)	−0.0013 (18)	0.011 (2)	0.009 (2)
C10	0.024 (2)	0.028 (2)	0.050 (2)	−0.0027 (15)	0.0077 (18)	−0.0040 (17)
C11	0.0243 (19)	0.033 (2)	0.055 (2)	−0.0011 (19)	0.0043 (19)	−0.0034 (18)
C12	0.037 (3)	0.034 (2)	0.065 (3)	0.0043 (18)	0.018 (2)	0.000 (2)
C13	0.049 (3)	0.058 (3)	0.040 (2)	0.010 (2)	0.000 (2)	−0.004 (2)
C14	0.049 (3)	0.046 (3)	0.038 (2)	0.015 (2)	0.0092 (19)	0.0030 (19)
C15	0.060 (3)	0.059 (3)	0.041 (2)	0.021 (3)	0.006 (3)	0.007 (2)
C16	0.024 (2)	0.033 (2)	0.063 (3)	−0.0032 (15)	0.0017 (18)	0.0051 (18)
C17	0.030 (2)	0.032 (2)	0.051 (2)	−0.0083 (17)	−0.0076 (19)	0.0090 (17)
C18	0.032 (2)	0.039 (2)	0.069 (3)	−0.0074 (19)	−0.008 (2)	−0.007 (2)
O4	0.035 (2)	0.079 (3)	0.158 (4)	−0.013 (2)	−0.012 (3)	0.078 (3)
N4	0.0195 (16)	0.0394 (18)	0.044 (2)	0.0012 (16)	0.0005 (16)	−0.0069 (15)
O5	0.0343 (18)	0.0569 (19)	0.0492 (18)	−0.0012 (15)	−0.0107 (14)	−0.0112 (14)
O6	0.066 (2)	0.0463 (18)	0.063 (2)	−0.003 (2)	0.002 (2)	0.0054 (16)
O7	0.065 (2)	0.0386 (18)	0.069 (2)	0.0065 (18)	−0.0138 (17)	−0.0132 (15)
N5	0.030 (2)	0.043 (2)	0.050 (2)	0.0103 (17)	0.0000 (17)	−0.0067 (17)
O8	0.106 (5)	0.204 (6)	0.119 (4)	0.114 (5)	−0.005 (3)	−0.032 (4)
O9	0.099 (3)	0.074 (3)	0.068 (2)	−0.049 (2)	−0.020 (2)	0.013 (2)
O10	0.085 (4)	0.063 (3)	0.157 (5)	−0.021 (2)	−0.065 (3)	0.043 (3)

### Geometric parameters (Å, °)

**Table:**

Zn1—O1	2.091 (3)	C7—H7B	0.99
Zn1—N3	2.128 (3)	C8—C9	1.512 (7)
Zn1—O2	2.135 (3)	C8—H8A	0.99
Zn1—N2	2.174 (3)	C8—H8B	0.99
Zn1—N1	2.179 (3)	C9—H9A	0.99
Zn1—O3	2.275 (3)	C9—H9B	0.99
O1—C11	1.439 (5)	C10—C11	1.500 (5)
O1—H1	0.855 (10)	C10—H10A	0.99
O2—C14	1.428 (5)	C10—H10B	0.99
O2—H2	0.844 (10)	C11—C12	1.516 (6)
O3—C17	1.429 (5)	C11—H11	1.00
O3—H3	0.849 (10)	C12—H12A	0.98
N1—C9	1.480 (5)	C12—H12B	0.98
N1—C10	1.489 (5)	C12—H12C	0.98
N1—C1	1.510 (5)	C13—C14	1.502 (7)
N2—C13	1.474 (6)	C13—H13A	0.99
N2—C4	1.508 (6)	C13—H13B	0.99
N2—C3	1.516 (6)	C14—C15	1.505 (5)
N3—C6	1.485 (5)	C14—H14	1.00
N3—C16	1.491 (5)	C15—H15A	0.98
N3—C7	1.508 (5)	C15—H15B	0.98
C1—C2	1.492 (7)	C15—H15C	0.98
C1—H1A	0.99	C16—C17	1.497 (6)
C1—H1B	0.99	C16—H16A	0.99
C2—C3	1.488 (8)	C16—H16B	0.99
C2—H2A	0.99	C17—C18	1.513 (5)
C2—H2B	0.99	C17—H17	1.00
C3—H3A	0.99	C18—H18A	0.98
C3—H3B	0.99	C18—H18B	0.98
C4—C5	1.513 (7)	C18—H18C	0.98
C4—H4A	0.99	O4—H4C	0.848 (10)
C4—H4B	0.99	O4—H4D	0.842 (10)
C5—C6	1.506 (7)	N4—O6	1.201 (4)
C5—H5A	0.99	N4—O7	1.242 (4)
C5—H5B	0.99	N4—O5	1.257 (4)
C6—H6A	0.99	N5—O9	1.193 (5)
C6—H6B	0.99	N5—O10	1.200 (5)
C7—C8	1.505 (7)	N5—O8	1.224 (6)
C7—H7A	0.99		
			
O1—Zn1—N3	155.21 (12)	C8—C7—H7A	108.0
O1—Zn1—O2	84.28 (11)	N3—C7—H7A	108.0
N3—Zn1—O2	98.67 (12)	C8—C7—H7B	108.0
O1—Zn1—N2	102.81 (12)	N3—C7—H7B	108.0
N3—Zn1—N2	101.87 (14)	H7A—C7—H7B	107.3
O2—Zn1—N2	78.08 (13)	C7—C8—C9	114.3 (4)
O1—Zn1—N1	77.96 (11)	C7—C8—H8A	108.7
N3—Zn1—N1	101.06 (13)	C9—C8—H8A	108.7
O2—Zn1—N1	160.27 (12)	C7—C8—H8B	108.7
N2—Zn1—N1	97.44 (14)	C9—C8—H8B	108.7
O1—Zn1—O3	80.11 (10)	H8A—C8—H8B	107.6
N3—Zn1—O3	75.89 (11)	N1—C9—C8	114.8 (3)
O2—Zn1—O3	82.88 (10)	N1—C9—H9A	108.6
N2—Zn1—O3	160.28 (12)	C8—C9—H9A	108.6
N1—Zn1—O3	102.22 (12)	N1—C9—H9B	108.6
C11—O1—Zn1	117.3 (2)	C8—C9—H9B	108.6
C11—O1—H1	109 (3)	H9A—C9—H9B	107.5
Zn1—O1—H1	123 (3)	N1—C10—C11	111.9 (3)
C14—O2—Zn1	117.0 (2)	N1—C10—H10A	109.2
C14—O2—H2	114 (3)	C11—C10—H10A	109.2
Zn1—O2—H2	121 (3)	N1—C10—H10B	109.2
C17—O3—Zn1	115.4 (2)	C11—C10—H10B	109.2
C17—O3—H3	111 (3)	H10A—C10—H10B	107.9
Zn1—O3—H3	124 (3)	O1—C11—C10	106.4 (3)
C9—N1—C10	109.4 (3)	O1—C11—C12	110.1 (3)
C9—N1—C1	106.5 (3)	C10—C11—C12	111.9 (3)
C10—N1—C1	108.3 (3)	O1—C11—H11	109.5
C9—N1—Zn1	113.5 (2)	C10—C11—H11	109.5
C10—N1—Zn1	101.7 (2)	C12—C11—H11	109.5
C1—N1—Zn1	117.1 (3)	C11—C12—H12A	109.5
C13—N2—C4	109.8 (4)	C11—C12—H12B	109.5
C13—N2—C3	106.4 (4)	H12A—C12—H12B	109.5
C4—N2—C3	110.4 (4)	C11—C12—H12C	109.5
C13—N2—Zn1	104.1 (2)	H12A—C12—H12C	109.5
C4—N2—Zn1	114.7 (3)	H12B—C12—H12C	109.5
C3—N2—Zn1	111.0 (3)	N2—C13—C14	113.3 (4)
C6—N3—C16	107.6 (3)	N2—C13—H13A	108.9
C6—N3—C7	108.1 (3)	C14—C13—H13A	108.9
C16—N3—C7	108.9 (3)	N2—C13—H13B	108.9
C6—N3—Zn1	111.0 (3)	C14—C13—H13B	108.9
C16—N3—Zn1	106.7 (2)	H13A—C13—H13B	107.7
C7—N3—Zn1	114.3 (2)	O2—C14—C13	104.8 (3)
C2—C1—N1	114.0 (4)	O2—C14—C15	111.7 (3)
C2—C1—H1A	108.7	C13—C14—C15	111.9 (4)
N1—C1—H1A	108.7	O2—C14—H14	109.4
C2—C1—H1B	108.7	C13—C14—H14	109.4
N1—C1—H1B	108.7	C15—C14—H14	109.4
H1A—C1—H1B	107.6	C14—C15—H15A	109.5
C3—C2—C1	112.8 (4)	C14—C15—H15B	109.5
C3—C2—H2A	109.0	H15A—C15—H15B	109.5
C1—C2—H2A	109.0	C14—C15—H15C	109.5
C3—C2—H2B	109.0	H15A—C15—H15C	109.5
C1—C2—H2B	109.0	H15B—C15—H15C	109.5
H2A—C2—H2B	107.8	N3—C16—C17	113.3 (3)
C2—C3—N2	116.7 (4)	N3—C16—H16A	108.9
C2—C3—H3A	108.1	C17—C16—H16A	108.9
N2—C3—H3A	108.1	N3—C16—H16B	108.9
C2—C3—H3B	108.1	C17—C16—H16B	108.9
N2—C3—H3B	108.1	H16A—C16—H16B	107.7
H3A—C3—H3B	107.3	O3—C17—C16	104.9 (3)
N2—C4—C5	117.0 (4)	O3—C17—C18	112.2 (3)
N2—C4—H4A	108.0	C16—C17—C18	111.0 (4)
C5—C4—H4A	108.0	O3—C17—H17	109.6
N2—C4—H4B	108.0	C16—C17—H17	109.6
C5—C4—H4B	108.0	C18—C17—H17	109.6
H4A—C4—H4B	107.3	C17—C18—H18A	109.5
C6—C5—C4	115.3 (4)	C17—C18—H18B	109.5
C6—C5—H5A	108.5	H18A—C18—H18B	109.5
C4—C5—H5A	108.5	C17—C18—H18C	109.5
C6—C5—H5B	108.5	H18A—C18—H18C	109.5
C4—C5—H5B	108.5	H18B—C18—H18C	109.5
H5A—C5—H5B	107.5	H4C—O4—H4D	108.6 (18)
N3—C6—C5	113.8 (4)	O6—N4—O7	120.6 (3)
N3—C6—H6A	108.8	O6—N4—O5	122.2 (3)
C5—C6—H6A	108.8	O7—N4—O5	117.2 (3)
N3—C6—H6B	108.8	O9—N5—O10	120.6 (4)
C5—C6—H6B	108.8	O9—N5—O8	118.4 (5)
H6A—C6—H6B	107.7	O10—N5—O8	120.2 (5)
C8—C7—N3	117.1 (3)		
			
N3—Zn1—O1—C11	77.9 (4)	N1—Zn1—N3—C16	128.7 (3)
O2—Zn1—O1—C11	176.2 (3)	O3—Zn1—N3—C16	28.7 (2)
N2—Zn1—O1—C11	−107.5 (3)	O1—Zn1—N3—C7	−77.0 (4)
N1—Zn1—O1—C11	−12.5 (3)	O2—Zn1—N3—C7	−172.1 (3)
O3—Zn1—O1—C11	92.4 (3)	N2—Zn1—N3—C7	108.3 (3)
O1—Zn1—O2—C14	104.3 (3)	N1—Zn1—N3—C7	8.2 (3)
N3—Zn1—O2—C14	−100.6 (3)	O3—Zn1—N3—C7	−91.8 (3)
N2—Zn1—O2—C14	−0.2 (3)	C9—N1—C1—C2	86.1 (5)
N1—Zn1—O2—C14	78.5 (5)	C10—N1—C1—C2	−156.4 (4)
O3—Zn1—O2—C14	−175.0 (3)	Zn1—N1—C1—C2	−42.3 (5)
O1—Zn1—O3—C17	−177.9 (3)	N1—C1—C2—C3	74.1 (5)
N3—Zn1—O3—C17	−4.1 (2)	C1—C2—C3—N2	−83.4 (5)
O2—Zn1—O3—C17	96.7 (3)	C13—N2—C3—C2	165.0 (4)
N2—Zn1—O3—C17	81.7 (4)	C4—N2—C3—C2	−75.9 (5)
N1—Zn1—O3—C17	−102.6 (3)	Zn1—N2—C3—C2	52.4 (5)
O1—Zn1—N1—C9	151.3 (3)	C13—N2—C4—C5	−141.8 (4)
N3—Zn1—N1—C9	−3.4 (3)	C3—N2—C4—C5	101.2 (5)
O2—Zn1—N1—C9	177.6 (3)	Zn1—N2—C4—C5	−25.1 (5)
N2—Zn1—N1—C9	−107.1 (3)	N2—C4—C5—C6	68.2 (6)
O3—Zn1—N1—C9	74.4 (3)	C16—N3—C6—C5	167.2 (4)
O1—Zn1—N1—C10	33.9 (2)	C7—N3—C6—C5	−75.3 (5)
N3—Zn1—N1—C10	−120.8 (2)	Zn1—N3—C6—C5	50.8 (4)
O2—Zn1—N1—C10	60.2 (5)	C4—C5—C6—N3	−85.8 (5)
N2—Zn1—N1—C10	135.5 (2)	C6—N3—C7—C8	148.8 (4)
O3—Zn1—N1—C10	−43.0 (3)	C16—N3—C7—C8	−94.5 (4)
O1—Zn1—N1—C1	−83.8 (3)	Zn1—N3—C7—C8	24.7 (5)
N3—Zn1—N1—C1	121.5 (3)	N3—C7—C8—C9	−74.6 (5)
O2—Zn1—N1—C1	−57.6 (5)	C10—N1—C9—C8	78.7 (4)
N2—Zn1—N1—C1	17.8 (3)	C1—N1—C9—C8	−164.5 (4)
O3—Zn1—N1—C1	−160.8 (3)	Zn1—N1—C9—C8	−34.1 (5)
O1—Zn1—N2—C13	−55.6 (3)	C7—C8—C9—N1	80.5 (5)
N3—Zn1—N2—C13	122.1 (3)	C9—N1—C10—C11	−174.5 (3)
O2—Zn1—N2—C13	25.6 (3)	C1—N1—C10—C11	69.8 (4)
N1—Zn1—N2—C13	−134.9 (3)	Zn1—N1—C10—C11	−54.1 (3)
O3—Zn1—N2—C13	40.8 (5)	Zn1—O1—C11—C10	−13.1 (4)
O1—Zn1—N2—C4	−175.5 (3)	Zn1—O1—C11—C12	−134.6 (3)
N3—Zn1—N2—C4	2.2 (3)	N1—C10—C11—O1	46.0 (4)
O2—Zn1—N2—C4	−94.3 (3)	N1—C10—C11—C12	166.3 (3)
N1—Zn1—N2—C4	105.2 (3)	C4—N2—C13—C14	73.0 (4)
O3—Zn1—N2—C4	−79.1 (5)	C3—N2—C13—C14	−167.5 (4)
O1—Zn1—N2—C3	58.5 (3)	Zn1—N2—C13—C14	−50.2 (4)
N3—Zn1—N2—C3	−123.8 (3)	Zn1—O2—C14—C13	−24.9 (4)
O2—Zn1—N2—C3	139.7 (3)	Zn1—O2—C14—C15	−146.3 (3)
N1—Zn1—N2—C3	−20.8 (3)	N2—C13—C14—O2	50.5 (5)
O3—Zn1—N2—C3	154.9 (3)	N2—C13—C14—C15	171.8 (3)
O1—Zn1—N3—C6	160.4 (3)	C6—N3—C16—C17	−173.2 (3)
O2—Zn1—N3—C6	65.3 (3)	C7—N3—C16—C17	69.8 (4)
N2—Zn1—N3—C6	−14.2 (3)	Zn1—N3—C16—C17	−54.0 (4)
N1—Zn1—N3—C6	−114.4 (3)	Zn1—O3—C17—C16	−21.4 (3)
O3—Zn1—N3—C6	145.7 (3)	Zn1—O3—C17—C18	−142.0 (3)
O1—Zn1—N3—C16	43.5 (4)	N3—C16—C17—O3	49.3 (4)
O2—Zn1—N3—C16	−51.7 (3)	N3—C16—C17—C18	170.7 (3)
N2—Zn1—N3—C16	−131.2 (3)		

### Hydrogen-bond geometry (Å, °)

**Table:**

*D*—H···*A*	*D*—H	H···*A*	*D*···*A*	*D*—H···*A*
O1—H1···O10	0.86 (1)	1.80 (1)	2.652 (5)	171 (5)
O2—H2···O4	0.84 (1)	1.80 (2)	2.624 (5)	166 (5)
O3—H3···O6	0.85 (1)	2.06 (2)	2.853 (4)	156 (5)
O3—H3···O7	0.85 (1)	2.54 (3)	3.199 (4)	135 (4)
O4—H4C···O8^i^	0.85 (1)	1.86 (4)	2.641 (7)	153 (8)
O4—H4D···O5^ii^	0.84 (1)	1.86 (2)	2.689 (5)	170 (8)
C9—H9B···O10^iii^	0.99	2.39	3.184 (6)	137

Symmetry codes: (i) *x*−1, *y*, *z*; (ii) −*x*+1/2, −*y*, *z*+1/2; (iii) −*x*+1, *y*+1/2, −*z*+1/2.
